# Becoming an Actionable Scientist: Challenges, Competency, and the Development of Expertise

**DOI:** 10.1007/s00267-023-01863-4

**Published:** 2023-08-11

**Authors:** Julia B. Goolsby, Amanda E. Cravens, Mary Ann Rozance

**Affiliations:** 1 Cooperative Institute for Research in Environmental Sciences, University of Colorado, Boulder, CO USA; 2 U.S. Geological Survey, Fort Collins Science Center, Fort Collins, CO USA; 3 University of Washington, Seattle, WA USA; 4 Present Address: U.S. Geological Survey, Forest and Rangeland Ecosystem Science Center, Corvallis, OR USA; 5 Present Address: Cascadia Consulting Group, Seattle, WA USA

**Keywords:** Actionable science, Usable science, Learning sciences, Competencies, Metacognition, Learning by doing

## Abstract

Demand has grown for actionable science to support real-world decision-making around climate change and related environmental management challenges. Producing actionable science requires scientists to hold a distinct set of competencies, yet relatively little is known about what these competencies are or how to train scientists to develop them. We conducted interviews with mid- and late-career scientists to empirically identify competencies they used when producing actionable science and to understand how they developed those competencies. We describe expertise in terms of 18 competencies—categorised as cognitive, interpersonal, or intrapersonal—that scientists integrated and applied to address the challenges associated with actionable science. We argue that scientists must engage in the social process of producing actionable science (i.e., learning by doing) to become an expert. Expert actionable scientists discussed the importance of learning through different contexts, processes, interactions, and relationships. By naming the competencies that constitute expertise, as well as methods for expertise development, our findings facilitate greater conscious awareness of the process of becoming an actionable scientist, a gradual process that starts during graduate training and continues as a career proceeds. Our results can inform the development of formal learning opportunities as well as the informal learning process that occurs whereby scientists take charge of their own learning.

## Introduction

Twenty-first-century environmental challenges are complex, demanding, and often intensified by the global impacts of climate change (IPCC 2018; Kareiva and Fuller 2016). Science can provide insights to support environmental managers and other decision-makers in responding to these challenges. However, scientific research, even when inspired by and relevant to real-world problems, is not always used by decision-makers and other non-scientific information users (“practitioners”). To address this problem, demand has grown for scientific outputs that practitioners will find not just useful but also usable when developing decisions, plans, and actions (Dilling et al. 2018; Bamzai-Dodson et al. 2021; Beier et al. 2017; Mach et al. 2020). This type of scientific output—as well as the processes by which it is produced and shared—has often been referred to as actionable science (Bamzai-Dodson et al. 2021; Filho 2015; Lemos et al. 2020; Mach et al. 2020; US EPA 2009; Argyris 1993) or usable science (Dilling et al. 2018; Dilling and Lemos 2011; Wall et al. 2017a). Actionable science has particularly received attention from federal agencies in the United States, who have viewed it as a means to ensure that public investments in science result in real-world impacts for specific decision-makers (e.g., NRC 2009, ACCCNRS 2015).

Producing actionable science involves a shift in the nature of a scientist’s work, as scientists must adjust their research methods, collaboration styles, and outputs to facilitate uptake in non-scientific settings (Bamzai-Dodson et al. 2021; Beier et al. 2017). Furthermore, they must balance more traditional scientific goals, such as theory building and novel knowledge generation, with the goals of practitioners (Dilling and Lemos 2011). Understanding practitioners’ goals and tailoring scientific outputs toward specific instances of real-world use requires closer collaboration with intended information users than traditional science (Pohl et al. 2017). Indeed, actionable science often involves coproduction, a method in which practitioners participate as partners in the scientific process (Meadow et al. 2015; Wall et al. 2017b; Bamzai-Dodson et al. 2021). Consequently, actionable science generally involves a more diverse set of practitioner interactions than traditional science and may demand additional competencies (i.e., skills and attitudes employed in a specific context) to manage those interactions.

Inter- and transdisciplinary (ITD) science, as well as the broader field of sustainability science, provide a starting point for understanding the competencies required to produce actionable science. ITD research (e.g., Knapp et al. 2019; Holden et al. 2019; Morse et al. 2007) is a research area whose definition remains contested (Pohl et al. 2017) but is generally considered to focus on the challenges and opportunities associated with integrating diverse perspectives in research, which is an important component of actionable science production. Guimarães et al. (2019) characterise ITD researchers in terms of their attitudes, skills, and motivations, including openness, reflexivity, creativity, and a desire to advance the common good. Fam et al. (2016) summarise the skills and dispositions of the transdisciplinary researcher as curiosity, commitment, critical awareness, creativity, communication, and connectedness. Sustainability science (Kates 2011) has a rich literature envisioning the ideal qualities of the sustainability professional who drives change toward sustainable futures (Wiek et al. 2011); within this broader category, the actionable scientist might be considered one type of sustainability professional, whose role is limited to supporting or informing action by others, rather than carrying the action out themselves (Mach et al. 2020). Brundiers et al. (2021) present a recent framework of seven competency domains for sustainability professionals, including implementation, strategic-thinking, values-thinking, futures-thinking, systems-thinking, interpersonal, and integrated problem-solving competencies. A similar effort by leaders of sustainability graduate programmes in the United States and Canada comprising the ANGLES network (“A Network for Graduate Leadership in Sustainability”) identified seven overlapping but different categories of aptitudes necessary for sustainability leadership: (1) fostering belonging, equity, diversity and justice; (2) building emotional intelligence; (3) collaborating for impact; (4) communicating and engaging; (5) strategic thinking and planning; (6) working productively and effectively; and (7) making work matter (Motzer et al. 2021). Comparison of actionable science competencies with those considered to be important for ITD and sustainability science can provide insight into whether the pedagogical approaches of ITD and sustainability are sufficient training to produce actionable science.

Translational research is another related scientific approach, originally developed in the biomedical domain, which focuses on addressing real-world challenges that would otherwise prevent biomedical science from becoming health treatments (Woolf 2008). With its focus on understanding practitioner experiences and building bridges across professional, disciplinary, and institutional boundaries, translational research shares similarities with both actionable science as well as ITD. Gilliland et al. (2019, p. 214) present what they call needed “basic traits of a translational scientist independent of his or her particular area(s) of expertise:” rigorous researcher, team player, boundary crosser, process innovator, domain expert, skilled communicator, systems thinker. However, these authors note, while “some of these characteristics may be found in practitioners of other scientific disciplines…the full complement as described here is arguably unique to translational science” (Gilliland et al. 2019, p. 214). The same insight applies to competencies for actionable science: it is not simply similarities or differences in individual competencies that bears consideration, but the overall assemblage of competencies taken together.

At present, demand appears to have outpaced the availability of actionable science expertise, evidenced by the growing number of programmes specifically aimed at enhancing researchers’ ability to produce actionable science (e.g., Association of Science and Technology Centres n.d.; Rozance et al. 2020; Smith Fellows n.d.) and by increasing attention to real-world impacts in funding solicitations (e.g., NASA 2022; NOAA 2023; NSF 2022). Yet there is a lack of clarity about the specific competencies demonstrated by those with expertise in actionable science. Researchers suggest many skills or competencies that may be needed to produce actionable science (e.g., Evans and Cvitanovic 2018; Mach et al. 2020; Wall et al. 2017b; Brunson and Baker 2016), some drawing on the literature from sustainability science competencies and pedagogy (Rozance et al. 2020) and others on translational science (Schwartz et al. 2017, Enquist et al. 2017). These publications are variable in their focus (from concrete skills to attitudes to personal traits), lack clarity in selection criteria, and many are case studies of training programmes that draw largely from the learning competencies in sustainability programmes, rather than actionable science in the real world (e.g., Rozance et al. 2020). An additional challenge is that much of the research on actionable science expertise to date is based on the personal research and mentoring experiences of actionable scientists within an author team (e.g., Ferguson et al. 2022; Rozance et al. 2020). Research team-derived descriptions of expertise may not align with definitions derived from broader, systematic investigation of actionable scientist experiences. A thorough investigation of actionable scientists in practice, what skills and knowledge they need to be successful, and how they developed those skills, can enrich the literature and practice of training actionable scientists.

Another gap in current research is that education or learning science scholars are rare within the author teams of publications describing skills for actionable science, as are academic teaching and learning specialists with experience in curriculum design and evaluation; the same is true even in adjacent fields such as sustainability science (Motzer et al. 2021). The learning sciences is an interdisciplinary field that integrates perspectives from cognitive science, educational psychology, anthropology, and computer sciences, among other disciplines, to study teaching and learning (Sawyer 2005). Research from the learning sciences can be used to more precisely describe the professional expertise needed for actionable science and provide insight into how such expertise is developed.

This paper addresses these gaps in the current literature by systematically investigating actionable science expertise through the lens of the learning sciences. We synthesise data from interviews with 27 experienced actionable scientists to describe 18 cognitive, interpersonal, and intrapersonal actionable science competencies and 15 challenges that contextualise the need for those competencies. We also report the process by which scientists developed their actionable science expertise. We hope this practical, in-depth account of actionable science expertise and its development can accelerate the process of becoming an actionable scientist.

## Theoretical Background: Developing Professional Expertise

An individual with expertise “has gained special skills or knowledge representing mastery of a particular subject through experience and instruction” (Ericsson 2014, p. R508). Etymologically, “expert” shares a root word with “experience” (Ericsson 2014), reflecting the intimate relationship between expertise and the learning experiences by which it is gained. Many learning theories have been used to identify what expertise is and how professionals (including scientists) develop it through experience (Hager 2011). There are two main perspectives on the nature of expertise and its development, one focusing on the individual learner, and the other on the learning context.

One view on expertise emphasises the “the rational, cognitive aspects of [professional] performance” (Hager 2011, p. 5) within the individual learner. Donald Schön, an influential early scholar of workplace learning, argued that professionals are “reflective practitioners,” developing increasingly sophisticated intuition through observation and reflection upon their own experiences (Schön 1987; 1992; also see Thorsen and DeVore 2013). Similarly, Ericsson and colleagues (1993) argue expertise develops through “deliberate practice,” or focused, structured efforts to increase skill level. Both scholars emphasise the ability to identify and solve domain-specific problems as a hallmark of mastery (Ericsson et al. 1993; Schön 1992). This cognitive perspective situates the development and practice of expertise within the individual, largely independent of context.

A second perspective on expertise emphasises that individuals work and learn within social settings, which shape their experiences and resultant expertise. According to this socio-cultural theoretical lens, learning happens by “actively engaging in the ongoing processes of workplaces” and performance is “significantly shaped by social, organizational, cultural and other contextual factors” (Hager 2011, p. 12). Expertise arises from one’s increasing capacity to negotiate situations and understand norms within a particular “community of practice,” and as such, expertise is inseparable from the context in which it develops (Billett 2001; Lave 1991, p. 63; Lave and Wenger 1991).

Billett (2001) integrates cognitive and socio-cultural perspectives, arguing that expertise emerges from the interaction between individuals and the social practices in which they participate, as individuals develop personal understanding of social experiences, which shapes how they participate. For Billett (2001), expertise arises as individuals refine their ability to respond not only cognitively, but also interpersonally and culturally, to ever-arising challenges. Billett (2008) also emphasises that individuals may experience the same situation differently depending on personal attributes and life histories. One important dimension of past experience is emotional intelligence and related personal capabilities. Mayer and colleagues (2016) characterise emotional intelligence as the ability to “(a) perceive emotions accurately, (b) use emotions to accurately facilitate thought, (c) understand emotions and emotional meanings and (d) manage emotions in [oneself] and others” (p. 291). Emotional intelligence supports a wide variety of professional capabilities (Goleman 1995; Rathore et al. 2017; Zeidner et al. 2004).

Integrating these perspectives suggests that professional expertise is multifaceted, consisting of cognitive and analytical abilities, interpersonal capabilities and ways of being in social situations, and intrapersonal attributes developed over time, such as emotional approaches to problem-solving. Furthermore, rather than a body of knowledge and capabilities that someone either possesses or does not, a holistic view recognises expertise as an integrated identity, with individuals becoming experts as they develop increasing facility in their area of specialisation and within their communities of practice (Hager and Hodkinson 2009; Lave and Wenger 1991).

The integrated nature of professional expertise suggests the need for integrated approaches to training and professional development (e.g., Billett 2001; Tynjälä and Gijbels 2012), such as occur in real-world settings. However, the burden of interpretation of real-world experiences need not lie entirely with the individual learner. Explicit awareness of the components of expertise, as well as the contexts in which professionals participate, can scaffold learning experiences to facilitate development of expertise. This paper strives to improve actionable scientists’ development process by identifying the components of actionable science expertise, as practised by experts, and pathways for its development.

## Methods

### Research Design

We operationalized our investigation of actionable science expertise by focusing on the specific competencies that comprise it, assuming this would make expertise easier to elicit and analyse. To do so, we drew upon the National Research Council’s report (NRC 2012), which examines a set of competencies and their development to describe how students might attain success in life and work (p. 1–2). As NRC (2012) argues, skills are “specific to—and intertwined with—knowledge within a particular domain” (p. 3). As such, we followed the NRC (2012) and the Organisation for Economic Co-operation and Development (OECD 2005) to select the competency as our unit of study, defined as “the ability to meet complex demands, by drawing on and mobilising psychosocial resources (including skills and attitudes) in a particular context” (p. 4). We also observe NRC (2012) to distinguish between cognitive competencies, “involving thinking and related abilities, such as reasoning, problem-solving, and memory” (p. 21); interpersonal competencies “used both to express information to others and to interpret others’ messages (both verbal and nonverbal) and respond appropriately” (p. 22); and intrapersonal competencies, which “[involve] emotions and feelings and [include] self-regulation—the ability to set and achieve one’s goals” (p. 21–22). After reviewing how the current actionable science literature describes competencies, we collected and analysed interviews with 27 scientists with reputations for actionable science expertise.

### Semi-structured Interviews

We conducted semi-structured interviews to investigate the research practices of actionable scientists who possess a significant degree of expertise. Study participants were all affiliated with the US Geological Survey Climate Adaptation Science Center (CASC) network, a leader in the production of actionable science within federal climate science (DeCrappeo et al. 2018), suggesting participants were likely to possess a high degree of expertise. Furthermore, since actionable science is an explicit CASC network priority, we expected these scientists were likely to be consciously aware of actionable science as a distinct approach. Measuring actionable science expertise is challenging in part because it may or may not be a conscious part of someone’s research identity, as scientists tend to identify with their core disciplinary field (e.g., wildlife biology). So, after we identified possible participants based on public web biographies on each regional CASC website, we consulted with CASC network leadership who are involved in awarding grants intended to fund actionable science projects in order to select participants with exemplary reputations for actionable science (Climate Adaptation Science Centers 2021). Recognising that expertise develops gradually, we chose mid- to late-career scientists (i.e., about five years or more into a permanent research or faculty role).

We invited 47 scientists distributed across the 8 CASC regions^1^ to participate in our study. After multiple email invitations and coordination with CASC leadership to achieve a balance of representation across regions, we interviewed 27 total scientists between July and October 2021 (see Table 1). Eight participants were social scientists, and 19 participants were natural scientists, most commonly ecologists (nine participants) or climate scientists (four participants). Participants reported having spent at least 40 percent and up to 100 percent of their career working on projects that involved both scientists as well as practitioners (e.g., resource managers, policymakers). All participants had many years of research experience, though seven participants at the time of interview had transitioned to managerial or boundary spanning roles. We did not explicitly collect demographic data, such as gender or race.

**Table 1:** Study sample by Climate Adaptation Science Center region

CASC Region	Invited	Responded^a^	Interviewed
Alaska	4	2	2
North Central	7	6	6
Northeast	6	2	1
Northwest	6	4	4
Pacific Islands	4	2	1
South Central	5	2	2
Southeast	8	7	5
Southwest	6	5	5

Note that the Midwest Climate Adaptation Science Center did not exist at the time of the study

^a^ Five scientists replied that they were too busy or misunderstood the request

Interviews followed a semi-structured interview protocol (see Supplementary Information: Interview Protocol) and lasted 50 min on average; we collected 22 h and 49 min of data. Participants were asked to describe their experiences conducting actionable science, including challenges and competencies used to overcome challenges. We also asked participants to describe how they developed these competencies, how they mentor others to do actionable science, and whether their perspective on actionable science expertise had shifted over time. Note that while we were interested in understanding competencies, the interview protocol referred to “skills and/or mindsets” as we presumed this would be more understandable for participants. To avoid confusion over the definition of actionable science as compared to other, similar terms, such as usable science, the first question asked how participants understood these terms and then the interviewer used the participant’s preferred term throughout the interview. Two members of the author team conducted the interviews, depending on availability.

### Data Analysis

Interviews were audio recorded with consent from participants and professionally transcribed. Transcripts were qualitatively coded in several rounds by one member of the author team in NVIVO Release 1.2 (426; QSR International). We first grouped interview data by key topics i.e., challenges, change over time, definitions of actionable science, development of expertise, specific actionable science projects, mentoring, and competencies. We then coded each topic individually, in several rounds.

Interview data describing challenges were open-coded in the mode of grounded theory (Corbin and Strauss 2008), allowing for themes to emerge that captured the breadth of participant experiences. We then grouped the challenges codes into broader themes for ease of interpretation, based on our knowledge of key challenges within actionable science (e.g., Beier et al. 2017; Ferguson et al. 2022; Mach et al. 2020; Rosemartin et al. 2023; Wall et al. 2017a, 2017b). We followed the same open coding process for interview data describing development of expertise. Given the preponderance of study participants who mentioned “learning by doing,” we recoded relevant interview data to identify diverse meanings of the phrase.

Due to the richness of the data, coding related to actionable science competencies evolved iteratively. First, we used emergent coding, following principles of grounded theory (Corbin and Strauss 2008), to capture participants’ own conceptualisations of expertise and its competencies, resulting in a total of 40 competencies. The first round of coding was informed by review of competencies described within the literature. We then recoded the data three more times, to simplify and refine this list; each round included significant discussion among our author team and consultation of relevant literature. This axial coding generated a final list of 18 competencies, which were then categorised as cognitive, interpersonal, or intrapersonal, following the NRC (2012) conceptualisation of the components of expertise.

## Results

### Actionable Science Competencies

**Fig. 1:**
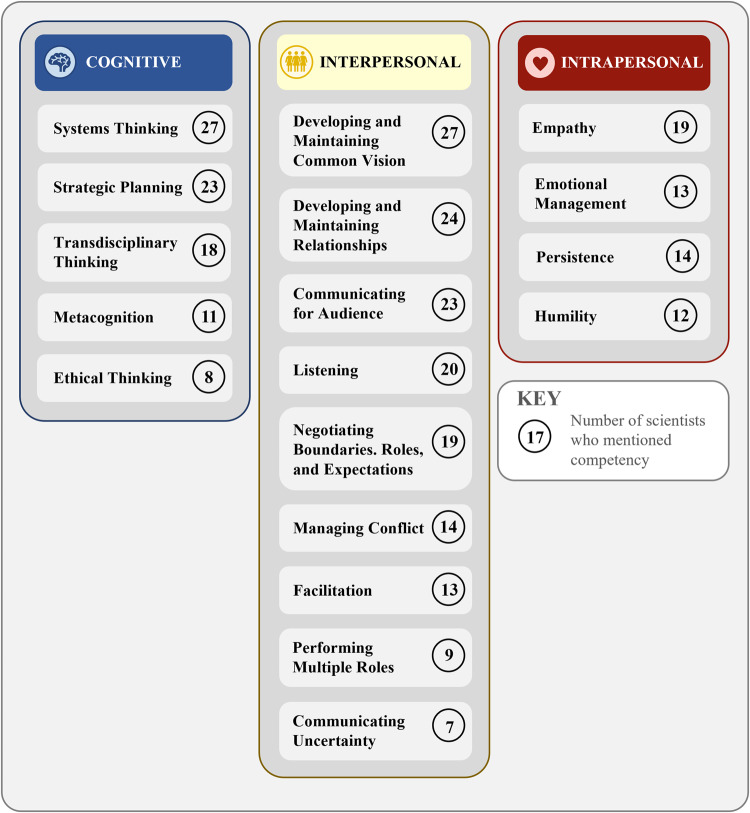
Cognitive, interpersonal, and intrapersonal competencies (for definitions see NRC 2012) described by scientists as important to their practice of actionable science, as well as the number of scientists who mentioned the competency

Scientists described 18 competencies they used to successfully produce actionable science. We classify these competencies into three domains: cognitive, interpersonal, and intrapersonal (see Fig. 1, Table 2).

#### Cognitive Competencies

Cognitive competencies involve “thinking and related abilities, such as reasoning, problem-solving, and memory” (NRC 2012, p. 21). Scientists described five cognitive competencies that helped them to analyse and process new knowledge, manage projects strategically, and think in new ways, among other pursuits (see Table 2). Systems thinking and strategic planning, both of which involve contextualising the project, were mentioned by the greatest number of scientists. Systems thinking was one of the two competencies mentioned by all scientists.

#### Interpersonal Competencies

Interpersonal or social competencies are “used both to express information to others and to interpret others’ messages (both verbal and nonverbal) and respond appropriately” (NRC 2012, p. 22). Given that actionable science is an inherently social process, it is not surprising that nine competencies mentioned by study participants fall into this category (see Table 2). Social competencies aided scientists’ ability to collaborate effectively, build trust, and manage relationships, among other goals. All scientists mentioned developing and maintaining common vision, and almost all mentioned developing and maintaining relationships and communicating for audience.

#### Intrapersonal Competencies

Intrapersonal competencies (i.e., emotional competencies) involve “emotions and feelings and [include] self-regulation—the ability to set and achieve one’s goals” (NRC 2012, p. 21–22). Not all scientists explicitly mentioned intrapersonal competencies, and we identified just four intrapersonal competencies within the interview data (see Table 2). Nineteen participants mentioned empathy, the highest in this category.

**Table 2:** Cognitive, interpersonal, and intrapersonal competencies (for definitions see NRC 2012) described by scientists as important to their practice of actionable science, as well as the number of scientists who mentioned the competency

Competency & Definition	Number of Scientists	Example Quote
**Cognitive Competencies**	27	
*1. Systems Thinking* involves thinking about how an actionable science project and its outcomes interact with the whole system or context in which the project is occurring, including history among partners or the organisations they represent, work culture and hierarchy, or other relevant cultural context, and politics. This also includes thinking about partners’ decision contexts and how the researcher themself fits into or interacts with partners and the larger system.	27	“The very first thing is knowing … that there are external constraints that shape what is and is not actionable. … [For example, during certain times of year, water managers in my project area have no] freedom to choose how to operate the dam. We could come up with all the most amazing information about streamflow predictions or new information for November, and we’d have to have an act of Congress for them to be able to use it, right? If we came up with the same information for streamflow in January, they could use it. … If you’re trying to improve management, you should look at the flexible period because that’s when [managers] can change things. … This is how understanding constraints can help direct you to science that can actually be useful, as opposed to science that ends up shouting in the void.” (S19)
*2. Strategic Planning* involves planning for the impact of current decisions on future events or designing a project backward from the desired outcome, as well as identifying the most feasible or effective partners, based on factors such as personality, background, and in the case of non-scientific partners, their potential to be champions for the project among practitioners. Strategic planning also includes thinking about future steps within the project, what current partners will need from the scientist once the project is over (e.g., updates to a data tool), and how a current project can lead to future projects.	23	“We’re not just throwing [our research] on the table and walking away. We’re actually thinking about, okay, here’s the science. Here’s how it’s going to come across to people. It’s going to have this effect or that effect or whatever in people’s thinking. Let’s think about what the rest of the world out there is really thinking about and what the stakeholders need, what’s important to them, and then we can figure out ways to move forward much more efficiently, really.” (S01) “I find it helpful, actually, to have a multi-year strategy with a lot of this, and you can’t predict the success of various projects being funded, but if one can daisy-chain a number of different projects that are related, then they feed back on themselves quite well—and these actionable science projects tend to benefit the largest group of people, when you can do that.” (S21)
*3. Transdisciplinary Thinking* involves thinking about a topic or question from multiple epistemological perspectives (e.g., academic discipline, indigenous knowledge, lived experience), which requires flexibility in thinking and an appreciation for multiple perspectives.	18	“I think that you need to be able to connect with and draw on others’ science. Science that you’re not necessarily an expert in, but you need to—so much of actionable science doesn’t fit in disciplinary boundaries or topical boundaries, or … what is needed doesn’t necessarily align with your expertise. You have to have an ability, a willingness to go beyond using the hammer that you know how to use and go figure out how to use the screwdriver instead. [Or work with others with expertise to] help you understand what it means in this other context.” (S19) “Curiosity, humility, that sense of value of other ways of knowing and other knowledges, like truly valuing them. That their knowledge is not more valuable, inherently, than other forms of knowledge. Valuing practical knowledge, local knowledge, indigenous knowledges.” (S14)
*4. Metacognition* is self-awareness of one’s own learning processes and how one self-regulates those processes in order to more efficiently learn from and recollect relevant experiences (Ormrod 2016). In practice, this can include self-awareness of one’s strengths and weaknesses, such as knowing the skills or knowledge one brings to the research team, as well as when it is best to rely on other team members. Metacognition can also include awareness of one’s own biases, which may influence one’s research and collaboration habits and decision-making.	11	“…to stop and really think through what’s happening in this process. What’s going well, what isn’t going well, to challenge your own biases and assumptions on an ongoing basis through a project…” (S06)
*5. Ethical Thinking* involves recognising the ethical dimensions of projects, such as whether and how research should be conducted so as to maximise benefits and reduce risks and negative impacts to research participants and collaborators. The Institutional Review Board (IRB) emphasises three foundational ethical principles, based on the Belmont Report (National Commission for the Protection of Human Subjects of Biomedical and Behavioural Research 1979): respect for persons, beneficence, and justice; these may guide interactions with stakeholders as well as research. When developing ethical research partnerships, Wilmer et al. (2021) propose “four principles: (1) appropriate representation, (2) self-determination, (3) reciprocity, and (4) deference, and two cross-cutting themes: (1) applications to humans and non-human actors, and (2) acquiring appropriate research skills” (p. 453).	8	“Communication, making sure community members felt good about the work that I was doing, that they were getting copies that they could use for their own purposes, that they’re adequately recognised in the work. When I presented the work, I tried to co-present if possible so they could share their personal experience…” (S24) “Why do we need to do … [the Institutional Review Board certification process]? Because people have treated others very, very badly in the past, and now we have to deal with that. [We have to consider] the legal and political frameworks surrounding working with indigenous populations. That there are particular standards that we have to meet when we work with indigenous populations. Then, the principles of those really apply to how we work with anybody.” (S06)
**Interpersonal Competencies**	27	
*6. Developing and Maintaining Common Vision* involves working with team members to develop a collective vision for the project, including defining objectives, ensuring objectives are useful to partners while advancing scientific aims of researchers, and scoping activities and projects. This skill also involves iteratively maintaining this vision among team members as a project progresses and responding flexibly to challenges encountered in a way that meets all team members’ goals and needs.	27	“You can’t come in with, ‘I think this is what they need,’ and start working on it, and go down that path. That just isn’t successful. We’re most successful when the products are really thought through from the very beginning with everybody in the room. The foundations, federal government partner, or private sector partner, whoever they are, that it starts at the very beginning of the conversation. I find that sometimes we try to get ahead of something and say, ‘Oh, I think this is going to be really important.’ Once in a while that works, but often you miss the mark and there’s not somebody there to catch what you do, and it doesn’t really connect or have the impact that you hope.” (S04) “Keeping the end use in mind and having the people there who would be the end users and working back from that, really pragmatically and theoretically, around how to get to the useful end product that people would actually use.” (S14)
*7. Developing and Maintaining Relationships* involves demonstrating a desire to form a longer-term relationship, developing trust, and then maintaining the relationship throughout a project and beyond. Developing and maintaining relationships involves demonstrating respect and curiosity through behaviours such as visiting partners in-person to learn about their context and remembering to check-in with partners.	24	“Being open to that slow building of trust and time. If you ask them a question, come back with an answer. If you’re going to take their time, then come back to them with, ‘Here’s what I did. Here’s what I heard, and here’s what I did. Does this help?’ Don’t just ask and never be heard from again. If you’re going to take the time, make it worth their time.” (S11) “[Continuing] to be in touch with [partners]. Following up. ‘You have this information. What’s your next step?’ We continue to be, through our … network, in really close long-term relationships with a lot of these government entities. We’re closely ingrained with what they’re doing. A lot of their planning, and regulatory, and policy work. It’s really a commitment to a long-term relationship with these different entities.” (S22)
*8. Communicating for Audience* involves adjusting the way the scientist communicates depending on who the audience is and what they need. Communication adjustments include changing the messenger, the mode of communication, and the content and form of the message, such as the level of detail. Some scientists also mentioned the importance of using humour to create a less formal atmosphere. Many scientists also emphasised the importance of connecting their message to relevant local contexts. Most scientists described communication as a two-way process; as such, scientists with this skill also described adjusting their message based on audience interactions.	23	“You pick and choose how you deliver messages to different audiences. If you’re trying to impose the perspective of an ecologist, in terms of what they think is important, onto a local audience, sometimes, I mean, you can tell pretty quickly when they begin to lose interest. I can tell you that. If you want to drone on and really put them to sleep, I guess that’s your choice. … [I]f I knew what that audience was, or thought I knew what that audience was, I would always try to gear my presentations to appeal to that particular audience. I think that’s part of the key, is trying to learn what would be important to your audience. Sometimes it surprises me because there would be times where you would start with maybe that general message, and there would be folks in the audience that would say, ‘Tell me more.’ You would go into it. You would peel that onion back a little more and go into a little more of the basic ecological detail. Sometimes that worked that way.” (S18)
*9. Listening* involves asking appropriate questions, remembering to ask questions, following up for clarification, and determining the right time and way to check in with collaborators such that they can provide their perspective while not overwhelming them with unnecessary detail. Some aspects of listening mentioned by scientists included asking open ended questions, creating venues for listening (e.g., workshops, regular check-ins), and doing background research to ensure sufficient context so that one asks questions in an informed way.	20	“Learning and knowing how to ask questions, how to ask follow-up questions, how to be listening really carefully for what somebody is saying and not saying. So that I’m understanding what they are getting, what they’re not getting, what might be unsaid or unstated that is maybe just they’re not—they’re not certain about it. They don’t feel comfortable asking.” (S06)
*10. Negotiating Boundaries, Roles, & Expectations* involves being able to adaptively negotiate the project timeline, project roles that are appropriate to ability and capacity, how project decisions are made, and who is involved in decision-making.	19	“Just making sure there’s an agreed-upon set of ground rules about who makes decisions, how we change decisions, who has power, who gets to be the author, who gets to be the co-author, how much work you get to contribute. Or you should need to contribute in order to be considered a co-author. … [A] set of ground rules about how we are going to work together, and who has ownership over which piece … Being really explicit about the expectations about the process and the project.” (S11)
*11. Managing Conflict* involves both avoiding or preventing conflict, as well as managing or resolving conflict when it does occur. One form of conflict resolution commonly mentioned by scientists was to remind people of a common goal or vision.	14	“Just a lot of a lot of talking. I don’t even know how else to put it. We had a just a lot of meetings and a lot of diplomacy. I really don’t know how to put it. It just had to be hashed out. … We had a shared mission and that part made it really easy, because we all wanted to do the right thing and have the right—well, right is a very value-laden word, but we all had the same goal in mind. That helped grease the wheels of getting us to consensus.” (S14)
*12. Facilitation* involves managing group processes effectively or knowing when to bring in an external facilitator to help do so. Facilitation involves tasks such as planning an achievable agenda, keeping meetings on-time, finding ways for group members who have not met to get to know each other, and implementing methods for collaboration (either analogue or digital). Facilitation involves also designing and implementing group structures and activities that support other competencies. For instance, group activities can be designed and managed in ways that enable teams to develop shared conceptions and goals or encourage group-level transdisciplinary thinking (Cravens et al. 2022).	13	“‘How are you going to elicit that kind of information in the process, and what do you need to be sensitive to, and what would be the best facilitation style to elicit that, and what should you be really transparent about?’ State it in the agenda or have a pre-meeting meeting just to talk about the ground rules.” (S03)
*13. Performing Multiple Roles*^a^ is the ability to adaptively perform multiple roles or functions within a team, across contexts, and as the project evolves, especially when a project has limited staff and funding.	9	“I think another skill is the flexibility in your training to do many parts of the chain of knowledge and information yourself. You need to be able to fill in for whatever else isn’t there, and we never have the ideal team to do the kinds of things that we want to do as perfectly as we want to do them. That means adapting on the fly.” (S10)
*14. Communicating Uncertainty*^b^ involves presenting complex scientific data, including its insights, caveats, and other limitations, in a way that makes sense to the audience and allows them to use the data appropriately. When effective, this usually takes a dialectic form, such that the communicator and audience reach a shared understanding. Several scientists also mentioned their use of visual communication aids.	7	“We were able to explain the uncertainty associated with it in a way that made sense and kept people from using it incorrectly. That gave them all the caveats. Thinking through all the caveats that you needed to warn them about without making them unusable because there’s so many caveats. Finding that place where both the managers felt it was useful, and the scientists felt it was robust. It was rigorous. That it was real science. It didn’t just get mushed into something that no longer meant anything and was going to be used incorrectly and more likely to give a bad result than an uncertain result.” (S07)
**Intrapersonal Competencies**	23	
*15. Empathy* involves actively trying to understand others’ lived experiences, without imposing one’s own experiences or views. Therefore, the practice of empathy also typically involves self-reflection and/or metacognition about one’s own experiences or views, to limit their influence on understanding others’ experiences.	19	“If you’re going to be in that world, you need to sort of think how they think and understand the pressures and responsibilities and limitations of their job. You need to be able to get into their head on some level and, sort of, understand what it is they’re looking for and where their sensitivities are, and what kind of pressures they face on the public or their bosses or their agencies.” (S25) “While you might feel like it’s your priority, it may not be somebody else’s priority, and so sometimes these projects take longer than you would expect because the other people are doing it on their time as they can.” (S16)
*16. Emotional management* involves noticing and regulating one’s own emotions. Among study scientists, one common way this manifested was remembering that negative interactions with team members and partners were not personal.	13	“I think, definitely learning to be able to let go, and move in a different direction, even though you’ve put a lot of effort into something. I think is something that you have to get used to.” (S04)
*17. Persistence* is the ability to continue with a project or activity despite setbacks. Many scientists who mentioned this competency also mentioned the importance of patience and flexibility, whether when dealing with partners’ schedules, slow progress on a project, or unexpected changes to the project.	14	“An ability to see things through even when they’re challenging and uncertain.” (S19)
*18. Humility* was described by scientists as approaching a project knowing they don’t know everything. Several scientists mentioned the importance of knowing that their knowledge is limited, and that science cannot achieve all goals, just like other ways of knowing have limits. This competency also involves admitting to oneself when one can’t do something.	12	“Walking into a room, saying—’I do bring some knowledge and expertise in this area. But I don’t know this area. I don’t know this region. I don’t know the dynamics of this community, but everybody in this room that I’m meeting with knows their little piece and their work so much better than I ever am going to. If I approach that—approach them as being experts, and that we all bring a piece of expertise to this—that combined is the only way this is moving forward.” (S06)

Competencies are organised by the number of scientists who mentioned them, in descending order. Quotes have been lightly edited for brevity, clarity, and anonymity, while maintaining content

^a^ Scientists described the performing multiple roles competency using the term “flexibility.” However, scientists also used that term to describe aspects of other competencies, such as being open to new ideas or project direction, thinking across disciplines or cultures, and adapting to unexpected changes in partner schedules or project timing. Therefore, we have elected to describe this competency as “performing multiple roles” to avoid confusion. Note that the frequent use of the term flexibility may be due to the fact that flexibility was used as an example in the interview protocol and thus may have inadvertently primed participants to mention flexibility more than they otherwise would have done (see Supplementary Information: Interview Protocol)

^b^ We chose to include the competency of communicating uncertainty, even though it might be considered a subset of the communicating for audience competency, because it was mentioned explicitly by so many scientists

### “Complex Demands:” The Cognitive, Interpersonal, and Intrapersonal Challenges of doing Actionable Science

**Fig. 2:**
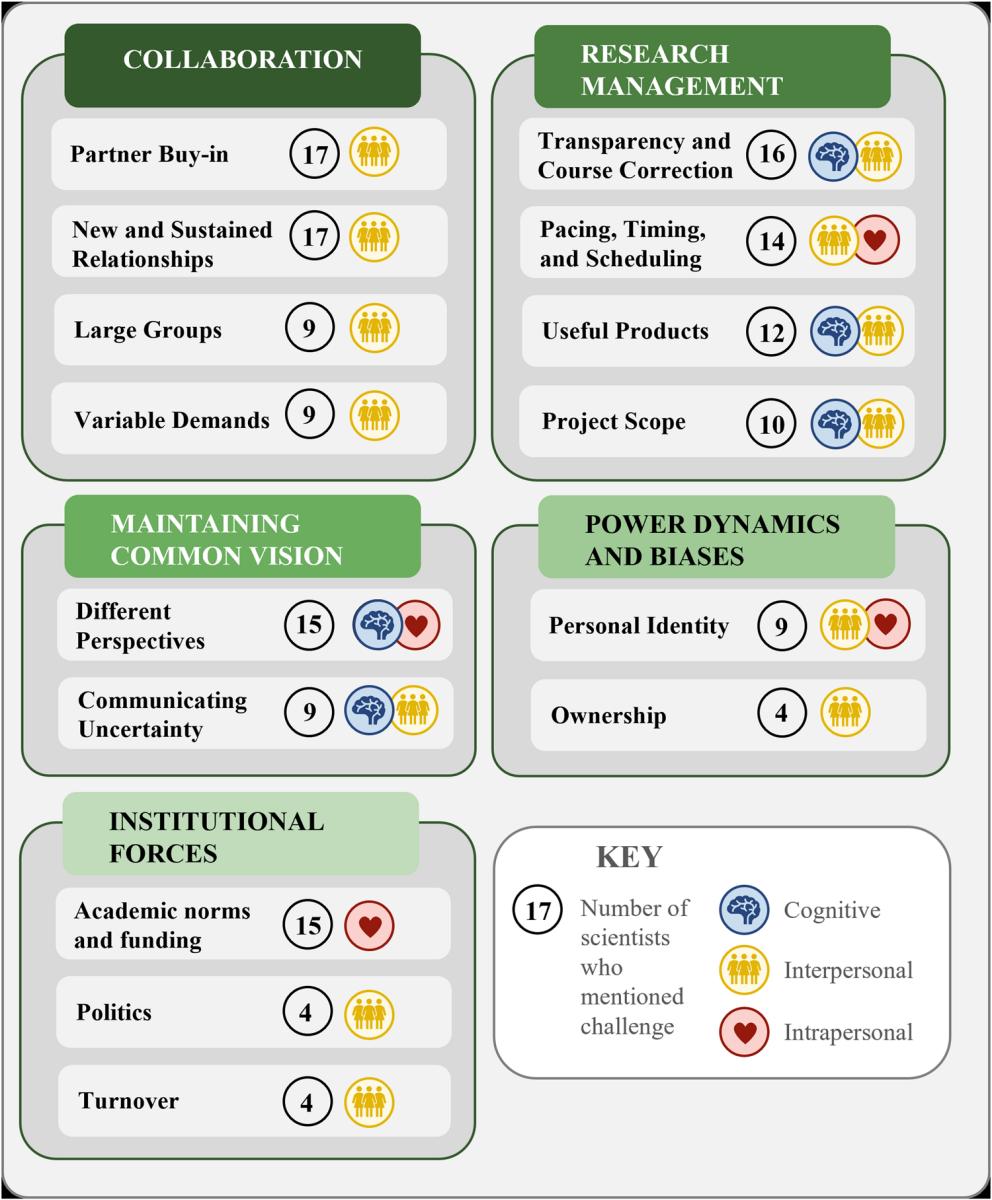
Challenges of actionable science, grouped by theme, including the number of scientists who mentioned the challenge and whether the challenge might be addressed via cognitive, interpersonal, and/or intrapersonal competencies

The OECD (2005) specifies that a competency is, “more than just knowledge and skills. It involves the ability to meet complex demands … in a particular context” (p. 4). The challenges of practising actionable science identified by scientists are one way to understand the contexts in which they practised, as scientists worked through the complex demands that these contexts placed upon them. Scientists identified 15 challenges associated with actionable science. Key challenges identified by scientists related to collaboration, research management, maintaining a common vision with partners, power dynamics and biases, and institutional forces (see Fig. 2). In Supplementary Table 1, we provide definitions for these challenges, as drawn from scientist description, as well as whether cognitive, interpersonal, and/or intrapersonal competencies might be used to overcome them. Challenges identified in this paper do not include those shared with traditional science approaches, such as data availability, unless scientists identified unique dimensions to those challenges in an actionable science context.

### The Process of Becoming an Actionable Scientist

Most scientists used the phrase “learning by doing,” or similar language, to describe their development of expertise. Scientists ascribed this phrase to a variety of learning methods, most commonly the iterative process of practice and reflection and the process of learning to participate in a community, but also peer-to-peer learning, apprenticeship, emulation of mentor behaviours, and on-the-job trainings (see Table 3). This plurality of definitions suggests that scientists did not see these methods of learning as separate, but rather as components of an integrated process that took place within the communities in which they worked. Indeed, scientists emphasised the importance of not just explicit and structured learning during actionable science projects, but also tacit and informal learning, especially to navigate social norms and other contextual factors (see Table 3).

**Table 3:** Methods of expertise development described by scientists in the study, as well as the number of scientists who mentioned each, and representative quotes

Method of expertise development	Number of Scientists	Example quote
*Peer to peer learning and mentorship.* Methods of learning through talking with peers and mentors, learning directly from peers or mentors, and emulating competencies as performed by experienced team members.	21	“Collaborating with social scientists in particular has probably been really important to my development, to be able to understand how you engage with what their needs are and how you can fit your science to it.” (S05) “I would say that’s the number one thing—learning from others, watching them do it, watching people who do it and do it well.” (S11)
*Participation.* A social learning process through which the learner develops an increased understanding of norms, social structure, language, and other socio-cultural contextual factors, such that they are able to function appropriately and contribute effectively within a particular community (Lave and Wenger 1991).	19	“[I joined] when I was 27 years old. I wasn’t taking charge right then, but I was introduced to folks, and person-to-person communication was key, and so you can start to participate in those conversations, and then build that trust that you’re working on things that they think are useful to help them accomplish their mission.” (S15)
*Reflection*. A method of competency development in which learners iteratively practice and reflect upon their experiences, including successes and failures, their own learning process, and their emerging identities (Dewey 1933; Thorsen and DeVore 2013).	13	“[Reflexive practice] asks the researcher to stop and really think through what’s happening in this process. What’s going well? What isn’t going well? To challenge your own biases and assumptions on an ongoing basis through a project.” (S06) “A lot of it has been learned the hard way. Not by somebody saying, ‘Just think about this,’ but by saying, ‘Oh, that didn’t work. How would I do that differently, or how could I modify that to have it work better?’” (S10)
*Formal learning experiences.* A discrete process of learning a competency, or aspect of a competency, through a workshop, training, or class provided while in school or during the learner’s career.	13	“I was very lucky to have a lot of communications training [from a science communication organisation] after grad school. That has been critical.” (S14) “So, 19–20 years old, I had somebody training me to do interviews with community members, viewing them as holders of knowledge.” (S06)
*Language development.* A process of learning language for actionable science, such that the learner can identify and interpret their experiences using the perspective and context that the language provides. Many scientists learned language by reading papers from peers.	10	“When it has a name, and there’s a community around it, you can think more deeply about it.” (S01)

Methods are organised by the number of scientists who mentioned them, in descending order. Quotes have been lightly edited for brevity, clarity, and anonymity, while maintaining content

None of the scientists in this study received formal training explicitly for actionable science. However, thirteen out of twenty-seven scientists reported that their formal learning experiences were transferrable to the practice of actionable science, including social science training in interviewing, degree programmes that taught systems or transdisciplinary thinking, and classes that challenged students to learn new skills quickly. Some scientists benefitted from classes that incorporated real-world projects, although their descriptions were not detailed enough to describe pedagogical style. Three scientists said that to successfully produce actionable science they had to actively overcome their academic training, such as an individualistic mindset or caveat-heavy communication style (S07; S14; S16). Even scientists with helpful formal learning experiences emphasised their need to refine expertise through practice.

It is important to note that scientists did not just “learn by doing” within study area or practitioner communities. They also learned to participate in boundary-crossing organisations (e.g., regional Climate Adaptation Science Centers) and the academic or research community. These communities shaped not just the methods by which scientists developed expertise but also what they learned. For example, the particulars of what each scientist learned about developing and managing relationships, such as trust building, navigating complex organisational hierarchies, or building on existing relationships, depended on knowledge embedded within a particular socio-cultural context.

Ten scientists described the development of language for actionable science, and related frameworks or ways of thinking, as critical to their development of expertise. Language facilitated their ability to reflect on their experiences and communicate with peers. Scientists learned language for competencies, learning processes, social processes, and actionable science itself, usually from academic publications and peers. One scientist with a natural science background emphasised the importance of learning from social science peers (S05). Actionable science publications also motivated some scientists by validating their actionable science research experiences.

Given that learning by doing requires an ability to persist despite repeated failures, almost all scientists emphasised that strong personal motivation is essential to the development of expertise— even in their current career stage. In fact, one scientist argued that a strong desire to work in the actionable science space is the only personal characteristic that drives success (S09). The most mentioned motivation for actionable science was what we name here a “service identity,” described by scientists as prioritising the interests and concerns of other team members and partners above their own. As one scientist (S14) described, “if you’re trying to be of service, you’re thinking about…who are the people who are going to use this? Who needs to be involved? How can we share in the making and in the sharing and in the rewards?” Another scientist described how a service identity motivates her work: “I think both in agencies and universities, we’re still in a place where the utility of what we do is measured in ways that don’t always meet up with doing this work. If those metrics are the way you consider yourself successful, in those metrics alone, then you may not be very happy doing this kind of work” (S10). Some developed this service identity through past life experiences, such as previous jobs or cultural upbringing. Regardless, scientists reported that their service identity improved both their motivation and success in actionable science projects.

## Discussion

### Awareness of Competencies and Context Facilitates Expertise Development

No matter one’s level of expertise, understanding what it means to have expertise can accelerate development. Naming competencies is a first step towards developing metacognition, or self-awareness, of one’s own expertise and learning processes (see Table 3; Ormrod 2016). A metacognitive perspective can be beneficial both for novices, who need to recognise the details of what actionable scientists do, and experienced researchers, for whom naming can lead to clarity in collaboration, mentoring, and curriculum development. There are parallels from the process of naming competencies in sustainability and the development of sustainability professionals (Brundiers et al. 2021; Brundiers et al. 2010; Kates 2011; Weik et al. 2011). Sustainability scientists went through a process of identifying and naming core competencies in order to establish university programmes to equip students with skills needed in sustainability professions (e.g., systems thinking, anticipatory thinking, normative skills, interpersonal skills, and strategic competence) (Weik et al. 2011), though did not always draw the expertise of the learning sciences and academic curriculum specialists as they did so (Motzer et al. 2021). Naming competencies in actionable science can support the personal self-awareness of actionable scientists and aid researchers in understanding pathways to develop their expertise over the course of a career. As a person moves beyond graduate training, they must learn to drive their own future professional development. Words for competencies that one seeks to develop can aid a researcher in identifying and finding institutional support for formal training options, finding appropriate mentors, and writing individual development plans (e.g., Vitae n.d.). By naming 18 distinct competencies derived from interviews with experienced actionable scientists and linking them to specific challenges those scientists experienced, we hope the present study contributes to the foundational practices of identifying competencies, the contexts in which they arise, and understanding the development of one’s own expertise.

Although the scientists in this study demonstrated deep expertise, it was challenging both to elicit and conclusively analyse into discrete competencies. The level of detail in scientist descriptions of each competency may provide some indication of scientists’ awareness of, or language for, different competencies, which has implications for mentorship and personal development. Competencies with broad definitions (e.g., developing and maintaining relationships) or that few scientists mentioned (e.g., ethical thinking) may benefit from increased attention from the actionable science community, particularly when mentoring earlier career researchers. Furthermore, few scientists directly described intrapersonal competencies but described many emotionally challenging obstacles, which suggests they employ more intrapersonal competencies than named. We believe the challenge of conclusively identifying competencies arises from the fact that scientists expressed their skills as experienced—not as discrete competencies, but rather as a general practice grounded within specific project contexts and their own identity.

### Expertise is Socially Integrated and Contextual

In this paper we present actionable science expertise in terms of 18 cognitive, interpersonal, and intrapersonal competencies (NRC 2012). Similarities and differences between the competencies we identified and those identified for adjacent fields (sustainability science, inter- and transdisciplinary science (ITD), and translational science) shed light on the particularities of actionable science as compared to these other approaches. In comparison to sustainability science, the actionable science competencies that we found place greater emphasis on social rather than cognitive processes; indeed, the need for greater attention to interpersonal skills has been noted within sustainability science (Brundiers and Wiek 2017). Illustratively, half of the 18 competencies were categorised as interpersonal competencies, focusing on actionable science as a way of creating science through social relationships with people and place. In comparison to ITD science competencies (e.g., Guimarães et al. 2019, Fam et al. 2016), which focus on bridging of ways of knowing within the research space rather than the use of research products in practitioner contexts, our results place more emphasis on practical competencies such as setting boundaries and expectations with practitioner partners. Also noted in the ITD literature is the “often-overlooked personal aspects” of transdisciplinary research (Sellberg et al. 2021, p. 301), which resemble the intrapersonal competencies we identified. Research on effective translational science traits emphasises the combination of deep domain knowledge with the ability to span boundaries and connect that knowledge to larger systems and innovative processes but places less emphasis on interpersonal skills than our results, other than being a team player and skilled communicator (Gilliland et al. 2019).

Among participatory practices related to actionable science (i.e., usable science, coproduction), we note that our interviews used terminology chosen by the scientist to acknowledge the importance of contextual expertise and facilitate scientists’ ability to describe their own expertise. However, we found little difference in the competencies described by scientists using these different terms, suggesting that while there are differences between actionable science and related participatory practices, there is little difference in the expertise needed to perform them.

Actionable science expertise is more than its component parts, a finding that has also been emphasised in sustainability science (Brundiers et al. 2021) and translational science (Gilliland et al. 2019). Past ITD research similarly has framed expertise holistically in terms of identity (e.g., Guimarães et al. 2019). Actionable scientists discussed how they contribute cognitive skills for project success but emphasised the integration of interpersonal and intrapersonal skills in driving the actionable science process. As Tynjälä and Gijbels (2012) note, “although the components of expertise can be analytically discerned, they are not to be seen as separate elements” (p. 207). Indeed, scientists described their expertise in an integrated fashion, using multiple competencies at once to address challenges unique to a project context. For example, one scientist (S04) described needing to adjust her expectations of outcomes as the project progressed, demonstrating interpersonal competency in developing and maintaining a common vision with her project partners, as well as the intrapersonal competency of emotional management.

In addition to using multiple competencies at once, scientists also demonstrated the same competency in different ways, depending on the context or challenges at hand. For example, transdisciplinary thinking among other academic researchers involved long, probing discussions with the goal of merging disciplinary perspectives, whereas scientists approached knowledge-bridging discussions with non-academic partners with humility and a desire to demonstrate respect at the forefront of their goals. In addition to demonstrating multiple forms of the same competency, scientists performed the same behaviour for different purposes. For example, scientists described visiting partners in person to demonstrate respect for lived experience, to better understand the project context, and to establish relationships.

It is important to note that we report the views of experienced actionable scientists. A fuller understanding of actionable science expertise may benefit from the views of practitioners. There is also a need for research that focuses more holistically on actionable scientists as people, with ways of being and knowing informed by diverse lived experience and personal characteristics such as career stage, gender, and socio-economic or racial background (Specht and Crowson 2022).

### Development of Expertise is Integrated into its Social Context

Half the competencies identified by scientists in this study point toward competencies that enable effective management of social processes within actionable science. This is perhaps because actionable science cannot exist without successful interpersonal interactions. Not only does actionable science typically focus on “wicked problems” (Rittel and Webber 1973) that cannot be solved from one professional or institutional perspective alone, but for scientific outputs to be actionable, they must be made with awareness of the larger social context in which they will be used, which requires input from those who live and work within that context.

Given its social nature, actionable science expertise is at least partly socio-culturally constructed, with competencies that must be developed within the context in which they are used and that exist as a set of norms between people, not just in one person (Lave and Wenger 1991). For example, one scientist (S01) had to learn to participate in an international science context, including developing sensitivity about who published which data: “I was really anxious to point out what was pretty clearly a negative impact … on the population. There was pushback from folks who said, ‘Well, that isn’t really our job. The species is really a Mexican species, and it should be—a paper like that should be authored by the Mexican biologist, not by the US biologist.’ I offered to help out with that, and then it never actually came to fruition. I understand that. That’s okay.” While some research has suggested that competencies for actionable science and adjacent approaches can develop through graduate training (e.g., Motzer et al. 2021; Brundiers et al. 2010), it is important that actionable scientists develop expertise within real-world contexts, so as to avoid, as Hanks (1991) describes in the foreword to Lave and Wenger’s seminal treatise on situated learning, 1991 “becom[ing] a master at managing the learning situation” (p. 21). This suggests that understanding the development of actionable science expertise requires drawing upon theories that explain how people learn in the workplace (Hager 2011). While graduate programmes could provide a solid foundation, we argue the development of actionable science expertise is a gradual process that starts during graduate training and continues for decades in the workplace.

Accordingly, scientists within this study provided an integrated perspective on expertise development, often described by interviewees as “learning by doing.” While their definitions varied, all scientists emphasised the importance of developing competencies within real-world contexts. Importantly, for scientists operating at the boundary of research and practice, learning occurs not just through interaction with practitioner partners, but also academic and cross-boundary communities. Scientists may benefit from increased awareness of both contexts as learning environments, including formal (e.g., graduate work) and informal or tacit learning (Marsick and Watkins 1990). These contexts may shape not just how an individual learns, but what they learn (Hager 2011). Additionally, scientists’ personal characteristics (such as gender) might interact with certain contexts or shape who most effectively develops particular competencies, though the design of our study did not allow us to fully explore these interactions.

Theoretical perspectives on expertise provide possible mechanisms for learning by doing in the workplace. Development of cognitive competencies, such as shifts in thinking patterns and greater awareness of the learner’s own thinking (i.e., metacognition), can happen via reflection on real-world professional practice (Schön 1987, Thorsen and DeVore 2013) and/or deliberate practice targeted at improving problem-solving (Ericsson et al. 1993). Interpersonal competencies can develop via participation in communities of practice (Lave and Wenger 1991). Both inter- and intrapersonal competencies can develop via conscious reflection with the goal of forming a personal understanding of real-world social situations (Billett 2001). Perhaps Hager and Hodkinson’s (2009) metaphor of “becoming” is the best way to think about the process of actionable science expertise development: participation in a particular learning context leads to personal development (i.e., competencies) and shifts in identity, all of which evolve iteratively as working relationships develop over the course of one’s career trajectory.

Scientists in this study noted the importance of motivation when managing the difficult task of bridging the goals or values of the scientific and practitioner communities they participated within. Scientists frequently mentioned a shift toward a service identity as an important way to understand their purpose within actionable science projects and to justify a set of career goals sometimes at odds with their own academic or government institutional context. As NRC (2012) states, “The rewards and meaning that people derive from becoming deeply involved in a community can provide a strong motive to learn” (p. 96). Research in adjacent fields has also identified the importance of motivation. For example, Guimarães et al. (2019) note that ITD scientists are not extrinsically motivated, but rather focused on the “common good” (p. 3) and are attracted to the field for its “transgressive” nature (p. 10). Scientists’ membership in other communities may support or hinder their development as an actionable scientist. For example, social science disciplines may already recognise the importance of some of the behaviours associated with actionable science (e.g., interview skills may teach active listening) while academic norms of communicating about statistical data and uncertainty may have to be unlearned to work effectively with practitioners. More broadly, since academic disciplines are a part of the context in which expertise develops, it is important to note that our results reflect the challenges and competencies deemed important by the scientists we interviewed and may not apply equally across all disciplines or scientific settings.

## Conclusion: Implications for Training Actionable Scientists

Doing actionable science requires significant interpersonal expertise and therefore develops as scientists learn through participation within specific communities and settings over the course of their careers. Actionable science expertise is therefore situated and can look different depending on the scientist. Not only did the scientists in this study employ different combinations of the 18 competencies described, but they integrated them into their work in different ways, depending on context and personal identity. Naming competencies, as we have done in this paper, can support the process of developing metacognition in both formal and informal learning settings. However, our results suggest that real-world learning experiences, which provide the opportunity for holistic engagement with actionable science, are the necessary foundation for expertise development.

Professional fields, such as sustainability, education, medicine, and law (Brayer 2012; Shulman 2005; Brundiers et al. 2010) offer a template for how graduate training programmes can support experiential learning. One example is the process of becoming a teacher (NRC 2010), in which students engage in graduated teaching exercises under the supervision of both an academic mentor and collaborating professional teachers (Orland-Barak 2016). In pedagogical terms, the student teachers’ behaviours and understanding of their behaviours are supported at each stage, with mentors lending the novices their own mental frameworks with which to interpret events and solve problems as they arise. By scaffolding experiences in this way, student teachers acquire the needed combination of competencies, develop metacognitive awareness to apply those competencies appropriately in context, and begin to perceive themselves as teachers (Ovens et al. 2016). Universities or mentors seeking to train actionable scientists can similarly design a series of experiences that support systematic, graduated engagement with real-world partners and projects, as is increasingly common within sustainability science programmes (e.g., ANGLES 2023; MITACS 2023). Sustainability programmes often utilise real-world learning models with problem-based learning, service learning, and internships or practicums in private and public sector organisations that strive for a real-world contribution (Brundiers et al. 2010), though the focus remains on training students and thus projects do not necessarily involve the sustained interaction with practitioners necessary to ensure use of research outputs.

While learning during graduate school can serve as the foundation for actionable science expertise development, truly becoming an actionable scientist happens over the course of a career, requiring the learner to take independent control of their learning process once they have completed a graduate degree. Steering one’s own professional development trajectory requires defining objectives for what one wants to learn (and often communicating those goals to others who might provide support, such as a post-doctoral supervisor, department head, or funding agency) and then identifying concrete learning opportunities that allow for the meeting of one’s goals. Metacognition is central to this process, as naming what is to be learned (i.e., competencies) and understanding contexts in which learning might happen are prerequisites to making decisions about one’s own future learning experiences. Some competencies (e.g., communication, facilitation) might be developed through professional development course offerings targeted at practising professionals; future research into the development of actionable science expertise may benefit from investigation of which competencies are best developed within formal versus informal learning settings.

Interpersonal and intrapersonal competencies can play a key role in a scientist’s transition from the type of learning that happens in graduate school to the self-directed learning that happens as principal investigator. Building relationships with others can aid in skill development, including seeking out mentors, participating in communities of practice, networking with other scientists, “reverse mentoring” where faculty learn from their students who might possess relevant community or practitioner expertise (Morris 2017), and so on. Actionable science often happens in teams, which means that a scientist might choose to consciously seek out collaborators with competencies that complement their own strengths; we note that even at advanced career stages not every scientist is likely to equally develop every competency discussed in this study. Being in charge of one’s own learning process represents a significant transition and often challenge, requiring emotional management and other intrapersonal competencies to aid in the transition. Reflective practice can be a key tool for emotional management as well as the development of other competencies. In their work based on a decade of teaching scientists to manage creative aspects of being a researcher, Ulibarri et al. (2019) similarly argue that intrapersonal attributes (i.e., self-awareness, mindfulness, and the emotional skill of self-compassion) are key abilities needed for scientists to take control of their own (in that case, creative) development and Cravens et al. (2022) highlight the importance of ongoing reflection in developing skill in science facilitation.

In summary, we hope that this learning sciences perspective on actionable science expertise and its development will aid scientists at multiple career stages hoping to produce science that addresses current challenges in environmental management. For scientists who share goals with those of actionable science, this paper may facilitate the development of terminology for expertise, thereby supporting the development of metacognition, and provide inspiration for their own development goals, as well as those of mentees. For universities and other research organisations, a better understanding of expertise provides insight into actionable science as a distinct approach to doing science, which may aid staffing investments, design of professional development opportunities, funding decisions, and the development of grant solicitations. We also hope that this description of actionable science expertise will be a starting point for other researchers to build upon and expand these findings.

### Supplementary Information

Supplementary Information

## Footnotes

1 The Midwest Climate Adaptation Science Center was created in 2021 and did not exist at the time of the study.
