# Supplementary material for: Becoming an Actionable Scientist: Challenges, Competency, and the Development of Expertise
Source: Environ Manage. 2023 Aug 11;72(6):1128–45. doi: 10.1007/s00267-023-01863-4 (PMC10570157; doi:10.1007/s00267-023-01863-4)
Supplement: Supplementary file 1 — Supplementary Information [file 267_2023_1863_MOESM1_ESM.pdf]

Supplementary Information for Becoming an Actionable Scientist: Challenges, competency, and the development of expertise by Goolsby JB, Cravens AE and Rozance MA

Journal: *Environmental Management*.

Corresponding Author: Amanda E Cravens, USGS Forest & Rangeland Ecosystem Science Center, aecravens@usgs.gov

Supplementary Table 1: Challenges of actionable science

Supplementary Table 1. Challenges of actionable science, the number of scientists who mentioned each, and whether challenges fall into the cognitive, interpersonal, and/or intrapersonal domain (for definitions, see section 4.2). A description of the challenge and a representative quote are provided. Quotes have been lightly edited for brevity, clarity, and anonymity, while maintaining content. Within each category, challenges are arranged in descending order based on the number of scientists who mentioned the challenge.

Theme<br><br>***Challenge and Description***	**Number of Scientists**	**Cognitive, interpersonal, and/or intrapersonal domain**	**Representative quote**
**Collaboration**			
1. Partner buy-in. Challenge of getting partners to fully buy into the project, perhaps when the project requires actions that are unfamiliar or new to them, or the research seems like a big commitment. This can remain a challenge throughout the research process.	17	Interpersonal	"Drawing [project partners] out and learning what questions to ask and being flexible in asking those questions and realizing … I was actually going to be … answering their questions … And just being okay with that." (S07)<br><br>"You can throw all the science you want at a conservation problem. If people aren't willing to go along with your recommendations, then it doesn't matter. Your actionable science dies on the vine because—maybe you get a nice paper published—but you're not actually going to … [achieve] that goal of affecting policy or human behavior if the humans that are affected by it aren't brought along as you go." (S01)
2. New and sustained relationships. Challenge of initiating and managing project relationships, especially given how many people can be involved in the project space and the long-term nature of many actionable science projects. Challenge of choosing project team members with compatible goals, style, or personality.	17	Interpersonal	"When you start getting long-term investments and stakeholder relationships and things like that, there's a different territoriality that can emerge there. It doesn't always look collaborative, [and] it can sometimes look conflicted, because people's livelihoods are based on those long-term investments and relationships. It becomes harder and harder to disentangle what is work, and what is personal." (S10)

Theme<br><br>*Challenge and Description*	**Number of Scientists**	**Cognitive, interpersonal, and/or intrapersonal domain**	**Representative quote**
3. Large groups. Challenge of working with large teams, including interpersonal dynamics, keeping the team informed, making sure everyone is at the table, and clashing personalities.	9	Interpersonal	“This was a project that had in it, an NGO. It had hungry regulators from multiple states. It had on the other end … [private] companies. As a stakeholder set that’s quite a challenging mix. This was not, ‘I’m working with a refuge manager.’ This was, ‘rooms of 20 people, some of them don’t get along, and some of them are direct financial competitors.’” (S09)<br><br>“We had a pretty big team, yeah. It was hard on the government end to keep all the relevant agencies together. … Keeping everybody informed, making sure that relevant parties are not left out.” (S22)
4. Variable demands. Challenge of balancing the variable demands of collaborative research, including when a researcher must perform many roles at once, or in close succession.	9	Intrapersonal	“I mean I was lead facilitator, moderator, and IT person. … It was too much, and … there were some costs involved in that. I wasn’t able to pay attention, in a way—to synthesize what I was hearing—because I was doing a lot of things at once.” (S08)
Research management			
5. Transparency and course correction. Challenge of involving partners in the scientific decision-making process, even though sometimes the decisions are highly technical, and ensuring the project continues to develop with partner needs in mind.	16	Cognitive<br><br>Interpersonal	“As researchers, we have this handicap, which is to move in a direction that is of supreme interest. It’s exciting. It’s interesting and we want go in that direction, but it’s always necessary to keep in mind that mission creep is a real thing. If you have a specific project, obviously, you have a list of deliverables, but following those most interesting things or one thing, but making sure that those align with what is within the stakeholder needs is another thing.” (S21)<br><br>“If you have a pretty good idea of a problem space, can you give [partners] a straw dog? Then are you being leading? That tension between providing something that helps move the conversation along and respects their time and something that isn’t so leading that you’re not respecting their input and their needs.” (S11)
6. Pace, timing, and scheduling. Challenge of accounting for limitations on how quickly the project can progress, whether due to the speed of the scientific process, limitations on non-scientific partners’ time,	14	Interpersonal<br><br>Intrapersonal	“Then all of a sudden, something that we were like, “Oh yeah, we think we’re going to get this back,” was taking months to get back. Part of that was just on us for not planning for that very well.” (S20)

Theme<br><br>*Challenge and Description*	Number of Scientists	Cognitive, interpersonal, and/or intrapersonal domain	Representative quote
misaligned schedules, etc. Challenge of working within partners' schedules and limited available time.			"We're trying to reconcile the partners hunger for product with the time it takes to make some primary scientific innovations." (S09)<br><br>"It's always hard to get manager's times. … They're just putting out fires, literally sometimes. … They don't have free time. They have three hours to talk to you." (S11)
7. Useful products. Challenge of determining what the most useful product is, perhaps for a range of audiences, and ensuring that the product has the maximum chances of being used.	12	Cognitive<br><br>Interpersonal	"If you're going to create some kind of tool—unless it slots directly into somebody's specific decision-making process—it's just another tool that gets lost in the millions of other tools that are available out there. … [However,] the more specific you get, the fewer people are going to use it, and the more it just tends to get lost." (S11)<br><br>"'Well, this person doesn't really make the decision. They just give information to XYZ person that makes the decision.' They're realizing they have to give information in a way that is useful to not just the person they're immediately working with but other people that are in their organization." (S26)<br><br>"Then, in the end, it feels like the hardest part is always—even if you've done the translational work really well—there still seems to be inertia or barriers to uptake." (S02)
8. Project scope. Challenge of defining the project scope, framing the project, identifying a need that is solvable with the people and tools at hand, and deciding how to divide up the work.	10	Cognitive<br><br>Interpersonal	"Taking the time to listen to what the managers say they need and want, and to recognize that that dialogue is challenging because you might be like, 'Well, what do you need?' and they're like, 'I don't know. What can you do?' That's a real struggle, and it has just to be an iterative process of showing them a little bit of what you can do, but maybe don't overwhelm them. Don't get too technical, but like, 'Here's a little bit about what we can do. Is that of interest? No? What else?' Then they can see, like, 'Oh, that's what you can do. Yeah, that's helpful, but no, I think what I really need is this.'" (S08)<br><br>"It took a while to figure out who was going to do the actual work and who was contributing ideas and feedback." (S19)<br><br>"We would get questions we simply could not answer, or the amount of data crunching would exceed our budget instantly. Every once in a while, I'd go to my

Theme<br><br>*Challenge and Description*	**Number of Scientists**	**Cognitive, interpersonal, and/or intrapersonal domain**	**Representative quote**
			climate science colleague, and just be like, "Can you do this?" He's just like, "Yeah, with a month and supe computer, I sure can." I was like, "Okay." We can't. We can't answer everything." (S06)
Maintaining common vision			
9. Different perspectives. Challenge of working with scientific and non-scientific partners with diverse and sometimes conflicting epistemological perspectives, due to different backgrounds (e.g., research, educational, cultural).	15	Cognitive<br>Interpersonal<br>Intrapersonal	"Now I'm going to take those same data and analysis … and try and package it for adaptation planning for … a tribe, and I'm realizing that it's just going to be fundamentally different: everything about the metrics I'm trying to pull out, the things that matter, the way it's presented, and also sort of the … nature of things, or how the resources they manage are operating on the landscape. It's already a really fundamentally different approach." (S05)<br><br>"[W]henever you're working just in interdisciplinary teams there's always challenges in just making sure that … what you say is actually understood, like what you're trying to communicate is actually landing on the other side." (S27)
10. Communicating uncertainty. Challenge of explaining uncertainty within the data—and the conclusions that can and cannot be drawn from data—to stakeholders who are generally less familiar with scientific uncertainty.	9	Cognitive<br><br>Interpersonal	"It can be challenging for communities when we come back with some more general statements. That's hard. That's where some of the narrative and the discussion comes in so that we really focus on some of the climate literacy side. Let me help you understand the power and limits of this data so that you're not over-reliant on it, and you're also not under reliant on it." (S06)
Power dynamics and biases			
11. Personal identity. Challenge of collaborating effectively with partners of similar or different identities (e.g., gender, race, culture, living in a rural vs urban setting). Challenge of becoming personally invested in a project. Challenge of managing negative views of scientific research and trying not to impose personal research perspectives on the project or otherwise disrupt ongoing processes.	9	Interpersonal<br><br>Intrapersonal	"I didn't think I spoke their language. I didn't think I wore their dress. I didn't think I read the same things. Once in a while, I would connect to somebody out there, but … I felt it in spades because I just didn't feel like I had the culture to relate to these people." (S25)<br><br>"It gets really hard for me to separate sometimes the personal versus the work parts of this, because I invest a lot of myself in what I do." (S10)

Theme<br><br>*Challenge and Description*	**Number of Scientists**	**Cognitive, interpersonal, and/or intrapersonal domain**	**Representative quote**
			"The stakeholders were very, very concerned about any hint that maybe the scientists were trying to be too prescriptive or tell them too much about things that they already knew better." (S05)
12. Ownership. Challenge of determining project ownership, including authorship and who owns the data and products.	4	Interpersonal	"There was a lot of issue that came up with who owns the data. … Some sensitivity was required for that." (S01)
Institutional forces			
13. Academic norms and funding. Challenge of achieving project funding, career advancement, and academic recognition while working on projects that conflict with the traditional norms, processes, and funding structures of the scientific community, particularly within academia, such as the pressure to produce academic papers. Obtaining funding for actionable science can be especially difficult due to longer timelines and co-produced outcomes that do not align with grant cycles and grant application requirements.	15	Intrapersonal	"There's a lot of folks out there who don't get credit, really, in their field for … [actionable science] work. … [T]here's still this underlying, sometimes, hesitation because if I do interdisciplinary work, it doesn't count. Spending time on an endangered species recovery panel means that's time away from writing a scientific paper or a grant proposal." (S01)<br><br>"We have these fantastic ideas but implementing them later on with stakeholders was probably not the best way to go, we realized, because we then encounter new ideas that we probably should've incorporated early on. So, making sure that stakeholder involvement, at whatever level, happens sooner than later. … [T]here's no funding mechanism in place to be able to do that. We're not funded, typically, to develop an idea. We're funded to bring an idea, or to take an idea that's well-developed and implement it." (S21)<br><br>"We get these grants for two years, three years, whatever it is, but I think this kind of work does take longer in many cases. Certainly co-production takes longer. It's hard to do on a short grant cycle unless you're getting a grant to do a project with a group that you already have those relationships established." (S01)
14. Politics. Challenge of navigating politics associated with the research due to the wider institutional and policy settings in which partners work.	4	Interpersonal	"Later on, with the project, some of those challenges with the politics and so on definitely did come up. I was really anxious to point out was pretty clearly a negative impact … on the population. There was pushback from folks who said, "Well, that isn't really our job. The species is really a Mexican species, and it should be—a paper like that should be authored by the Mexican biologist, not

Theme<br><br>***Challenge and Description***	**Number of Scientists**	**Cognitive, interpersonal, and/or intrapersonal domain**	**Representative quote**
			by the US biologist.” I offered to help out with that, and then it never actually came to fruition. I understand that. That’s okay. That’s the other piece. When you’re doing your own science and your own lab and you’re internally focused, you get to make the decisions about the research and about how the research is disseminated and so on. When you’re doing, I think, at least some kinds of applied science, maybe all kinds, you have to recognize that you don’t get to call all the shots.” (S01)
15. Turnover. Challenge of managing the project when one collaborator switches jobs partway through the project.	4	Interpersonal	“[Y]ou might start working with somebody [in an agency] who’s really psyched up about climate stuff, and then they leave and somebody else comes in and says, ‘Sorry, low priority.’” (S03)

Supplementary Information: Interview Protocol

Block 1 – Your understanding of and experience with actionable science

1. A number of terms get used to describe a roughly similar concept: science that is actionable and focused on the needs of stakeholders. These terms include usable science, actionable science, coproduction, translational ecology, etc.
    a. Which of these terms are you familiar with?
    b. Do you prefer one of these terms and why?
    c. How would you define actionable science*?
2. When and how did you start doing actionable science?

    Prompt <u>if needed</u>: Why did you get interested in it?

    Prompt if needed: How long have you been doing actionable science projects?

3. Approximately what percentage of the research time over your career have you spent doing actionable science projects?

    Prompt <u>if needed</u>: You've already said it's a [high/moderate/low] percentage, but if you were to give a number…

Block 2 – Actionable science project example

Now, in order to ground the discussion, we would like to ask you to think of one specific actionable science project. It would be great if you could please choose a project that is complete and has results you have shared in the world, and preferably a project you consider to be successful. This could be the project you consider most significant, or simply your most recent project. I'll give you a second to think about it – let me know when you have a specific project in mind.

Great, thank you.

4. Can you tell me the project name and a bit about it?
5. What challenges or hiccups related to actionable science did you encounter when doing this project?
    a. *Prompt <u>if needed</u>: Did you encounter any challenges at the start of this project? With initial development of the idea?*
    b. *Prompt <u>if needed</u>: Did you encounter any challenges when putting together the research team? Did they have different backgrounds? How did they get involved?*
    c. *Prompt <u>if needed</u>: Did you encounter any challenges when developing research questions and a plan?*
    d. *Prompt <u>if needed</u>: Did you encounter any challenges working with your team through the data collection and data analysis phases?*
    e. *Prompt <u>if needed</u>: Did you encounter any challenges related to producing or sharing the results of the project? Who was the intended audience?*
6. What skills, mindsets or techniques did you have to draw on during this project to ensure a successful outcome?

Block 3 – Reflecting on [Actionable Science]

7. In general, beyond just the project you described, are there any other important mindsets or skills a scientist needs to have to do actionable science?

    Prompt <u>if needed</u>: For example, being flexible or communicating clearly or asking open ended questions are skills.

Prompt if needed: I heard you mention [X,Y,Z skills] when describing [project example]. Would you consider any of those to be more important?
Prompt if needed: Can you tell me more? Could you give me an example of how you used [skill]? What did it look like to use [skill]? What do you mean by [skill]?

8. How did you develop your current skills or mindsets for doing actionable science?

    Prompt if needed: In school, formal training, through experience, working with peers, etc?

9. When you are mentoring your students to do actionable science, what skills or mindsets are you trying to cultivate in your students?
    Prompt if needed: If you haven't mentored students, what skills or mindsets would you cultivate?
    Prompt if needed: How do you go about cultivating these skills?

10. Has your perspective on what it takes to do actionable science shifted over time?
11. Is there anything else you want to tell me?

** The interviewer asked the participant their preferred term related to actionable science and replaced actionable science with that term.
